# The efficacy and safety study of electro-acupuncture for severe chronic functional constipation: study protocol for a multicenter, randomized, controlled trial

**DOI:** 10.1186/1745-6215-14-176

**Published:** 2013-06-15

**Authors:** Zhishun Liu, Jia Liu, Ye Zhao, Yuying Cai, Liyun He, Huanfang Xu, Xiaohua Zhou, Shiyan Yan, Lixing Lao, Baoyan Liu

**Affiliations:** 1 GuangAn Men Hospital, China Academy of Chinese Medical Sciences, No. 5, Beixiange street, Xicheng District Beijing 100053, China; 2 China Academy of Chinese Medical Sciences, No. 16, Dongzhimen Nei Nanxiaojie, Dongcheng District, Beijing 100700, China; 3 Department of Biostatistics, University of Washington, Office H655E, HSB Box #357232, Seattle, WA 98198, USA; 4 Center for Integrative Medicine, University of Maryland School of Medicine, East Hall, 520 W. Lombard Street, Baltimore, MD 21201, USA

**Keywords:** Chronic functional constipation, Electro-acupuncture, Multicenter RCT, Efficacy, Safety

## Abstract

**Background:**

Previous research has shown that electro-acupuncture (EA) may be effective for functional constipation. We report a protocol for a randomized controlled trial using EA to confirm the efficacy and safety for severe chronic functional constipation.

**Methods/design:**

This is a randomized, controlled trial. A total of 1,034 patients will be randomly allocated into the EA group (n=517) and the sham EA group (n=517). The EA group receives needling at ST25, SP14 and ST37 and the sham EA group receives needling at sham ST25, SP14 and ST37. The primary outcome measure is the changed number of weekly average complete spontaneous bowel movements(CSBMs) during 8 weeks of treatment, compared with baseline. The secondary outcome measures are: 1) the proportion of participants having three or more CSBMs on average per week; 2) the changed number of weekly average CSBMs during weeks 9 to 20; 3) the changed number of weekly average spontaneous bowel movements during 8 weeks of treatment; 4) stool consistency; 5) degree of difficulty in defecation; 6) patient assessment of constipation quality of life questionnaire (PAC-QOL); 7) incidence of adverse events; and 8) usage of medicine for constipation.

**Discussion:**

This trial will evaluate the efficacy and safety of EA for severe chronic functional constipation.

**Trial registration:**

Protocol Registration System of ClinicalTrial.gov, NCT01726504

## Background

Constipation is a common gastrointestinal disease. Recent data showthat the incidence of chronic functional constipation is 14.7% in the USA [1] and 11.6% in Asia [2]. Despite an instant effect, long-term use of western medicine usually results in drug dependence, melanosis coli, laxative constipation or even canerization [3]. To date, there are hardly any specific therapies for chronic functional constipation. Systematic reviews indicate that acupuncture is probably effective for the disease, which maybe better than western medicine, but the evidence is limited [4]. Our pilot study showed deep needling electro-acupuncture (EA) worked from week 1, which was quicker than either shallow needling (worked from week 2) or medication with lactulose (Duphalac) [5,6]. The treatment group adopted deep needling at ST25, who showed a frequency of weekly defecation of 2.75±1.48, compared with the shallow needling group (0.79±0.93) and the medication group (2.17±1.15) after 4 weeks of treatment. This study indicated that deep needling EA at ST25 might be superior to shallow puncture and Duphalac, but further research is needed to provide more evidence.

## Methods/design

### Design

**Figure 1:**
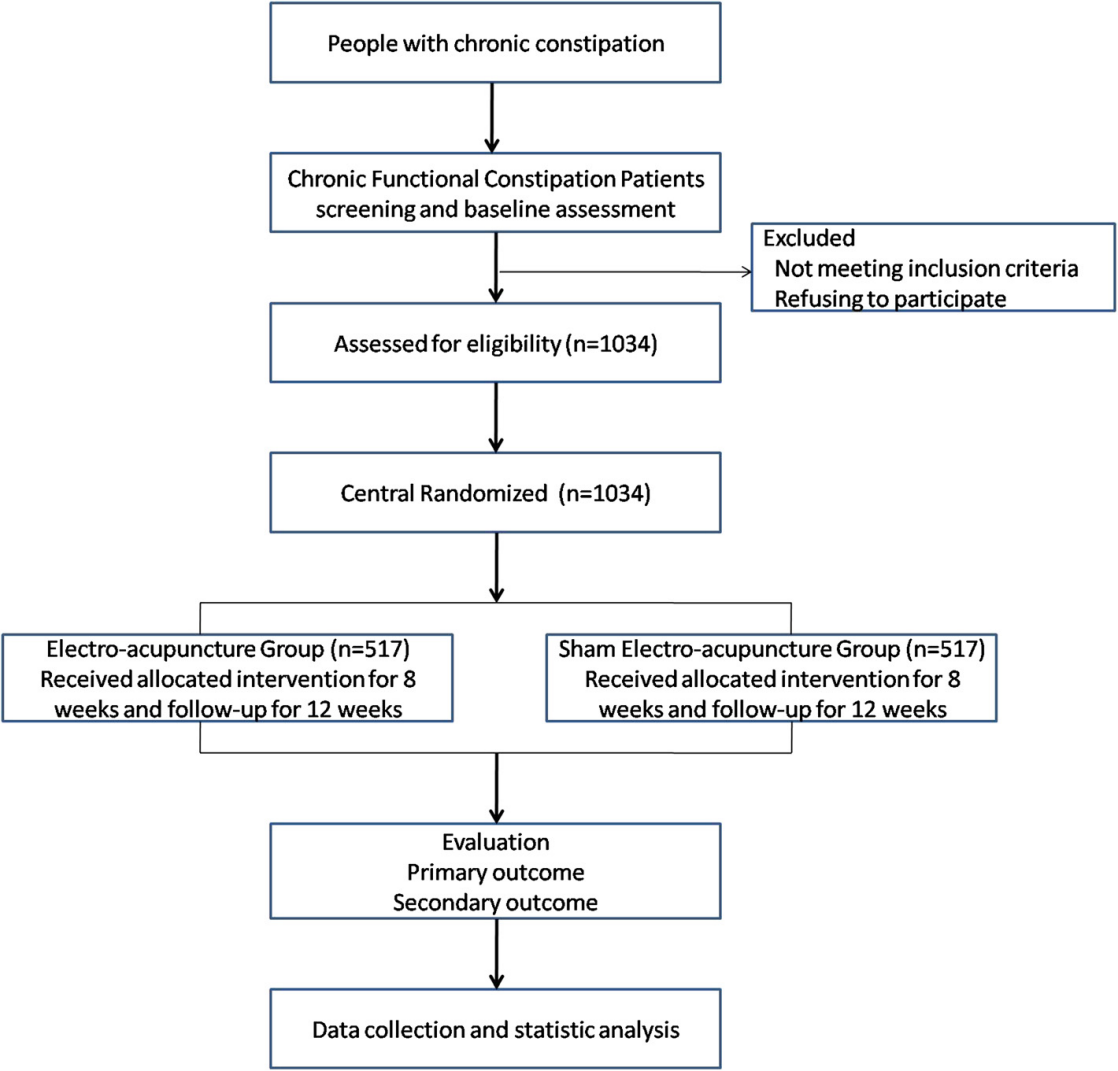
Flow diagram of study design.

This is a randomized, controlled, two-arm, large-scale trial comparing EA with sham EA (Figure 1). Guang An Men Hospital of China Academy of Chinese Medical Sciences is the investigative hospital, and 1,034 patients will be included from the following fifteen hospitals: Guang An Men Hospital of China Academy of Chinese Medical Sciences (South Branch); West China Hospital of Sichuan University; Guangdong Hospital of Traditional Chinese Medicine (TCM); Heilongjiang Institute of TCM; Beijing Hospital of TCM; Huguosi TCM Hospital of Beijing University of TCM; 1st Affiliated Hospital of Tianjin University of TCM; Dongzhimen Hospital of Beijing University of TCM; 3rd Affiliated Hospital of Zhejiang University of TCM; 301 Hospital; Wuhan Hospital of Integrated TCM and Western Medicine; Yueyang Hospital of Shanghai University of TCM; Nanjing University of TCM; Jiangsu Hospital of TCM; Anhui Hospital of TCM. All acupuncture manipulators are required to have an official license and individual clinical work experience lasting more than 2 years.

The participants will be allocated through central randomization in a 1:1 ratio. The central randomization will be performed by the Clinical Evaluation Center at the China Academy of Chinese Medical Sciences. A random number and group assignment will be immediately received by telephone, mobile phone, or website sent from the central randomization system.

The duration of this study is 22 weeks for each participant. All participants are asked to record constipation diaries from 2 weeks before randomization for the baseline data. If they are eligible for this trial, they will be asked to continue the diaries, recording from weeks 1 to 20 after randomization.

Participants will receive 28 sessions of EA or sham EA over 8 weeks, with a treatment frequency of five times per week in the first 2 weeks, and three times per week for the following 6 weeks. Each session will lasts for 30 minutes. The follow-up period is 12 weeks (weeks 9 to 20 after randomization).

### Patients

#### Study population

This study will include patients with severe chronic functional constipation (n=1034). To ensure the precision of the results of this study, we developed the following eligibility criteria.

#### Inclusion criteria

Participants will be included if they fulfil the following conditions: meeting the diagnosis of Rome III criteria [7] for chronic functional constipation; severe chronic constipation (two or fewer complete spontaneous bowel movements (CSBMs) per week for more than 3 months; aged 18 to 75 years old; no use of medicine for constipation during the 2 weeks before enrollment (except rescue medication usage which is defined as follow: if participants do not have a bowel movement for three or more consecutive days during the trial, they were permitted to take 110 ml Glycerol Enema, H11022362110, Beijing Maidihai Medical Co. Ltd, Beijing, China; 110 ml/bottle); no acupuncture treatment for constipation in the previous 3 months; never joined any other trial in progress in the previous 3 months; volunteered to join this research and signed the informed consent.

#### Exclusion criteria

Patients with any of the following conditions will be excluded: irritable bowel syndrome and secondary constipation caused by endocrine, metabolic, nervous or postoperative diseases or drugs; constipation accompanied by serious cardiovascular, hepatic, renal, or psychiatric diseases, cognitive dysfunction or aphasia, or severe dystrophy affecting the cooperation for examination or treatment; pregnant women or women in lactation; constipation accompanied by abdominal aneurysm, hepatosplenomegaly, *etcetera*; bleeding disorders, or regular anticoagulant drug users, such as warfarin and heparin, *etcetera*; cardiac pacemaker carrier.

#### Recruitment procedures

Participants will be recruited by newspaper, television, internet propaganda (http://www.gamhospital.ac.cn/) and posters in the community and hospitals (both TCM hospital and western medicine hospital). Prospective participants will be asked to talk face to face with the study coordinator to discuss the study and provide information regarding eligibility criteria. If the patient is eligible and interested in participating, they will be invited for a baseline screening visit after diagnosis by the doctor of the anus-intestines department or the gastroenterology department.

#### Ethical considerations

This trial has been approved by all fifteen local ethics committees (September 6, 2012) including: Guang An Men Hospital of China Academy of Chinese Medical Sciences (South Branch); West China Hospital of Sichuan University; Guangdong Hospital of TCM; Heilongjiang Institute of TCM; Beijing Hospital of TCM; Huguosi TCM Hospital of Beijing University of TCM; 1st Affiliated Hospital of Tianjin University of TCM; Dongzhimen Hospital of Beijing University of TCM; 3rd Affiliated Hospital of Zhejiang University of TCM; 301 Hospital; Wuhan Hospital of Integrated TCM and Western Medicine; Yueyang Hospital of Shanghai University of TCM; Nanjing University of TCM; Jiangsu Hospital of TCM; Anhui Hospital of TCM. The trial follows the principles of the Declaration of Helsinki (Version Edinburgh 2000). Before the randomization, all patients will be requested to sign a written informed consent; meanwhile, they will be given enough time to decide whether they are willing to participate in this trial, or choose other treatment options.

#### Randomization

A central stratified block randomization will be designed for this study by the central randomization system with a 1:1 allocation ratio. Stratified randomization will be made in terms of center. The randomization protocol and related index are produced by professional biostatisticians with SAS 9.3 software, procedure PROC PLAN (SAS software (Beijing) Co., Ltd, Office 1801-1803, E1 building, Dongfang Square, No. 1 Changan Road, Dongcheng District, Beijing, China, 100738). Related content such as the setting parameters will be saved with the blind codes. The randomization will be conducted by a central randomization system (telephone version and internet version). If a volunteer meets the inclusion criteria, the appointed center researcher will apply for the randomized number.

The randomization protocol will be designed by a statistician of the China Academy of Chinese Medical Science who will not be involved in the later statistical work of this project.

Blind codes mean the randomization protocol and the parameters are set during the procedure. The randomization protocol designer will seal these files and sign them. The sealed files will be deposited by an appointed member of staff who will not take part in this project. Central randomization has strict limits of authority; no one can check the files except the top principal investigator.

### Interventions and comparison

#### Acupuncture group

The acupuncture points are Tianshu (ST25), Fujie (SP14), Shangjuxu (ST37) (Additional file 1: Picture 1).

After sterilizing the skin, filiform needles are inserted 3 to 8 cm into bilateral ST25 and SP14 vertically and slowly without any manipulation until touching the parietal peritoneum. The standard for reaching the parietal peritoneum is that the participant feels sharp pain again (after the first pain of piercing the skin) and, meanwhile, the manipulator feels resistance from the needle tip. An electric stimulator (SDZ-V EA apparatus; Huatuo, Suzhou, China) is applied to bilateral ST25 and SP14 with a dilatational wave of 10/50 Hz and electric current between 0.1 and 1.0 mA. The participant's abdominal muscle twitching mildly indicates the appropriate dose. The bilateral ST37 is inserted 3cm - twirling, lifting and thrusting three times. A local sour and heavy feeling indicates the appropriate dose. Steady small twirling, lifting and thrusting manipulation will be used three times in each session.

Each session will last for 30 minutes per day. The participants are treated continuously for 8 weeks. During the first 2 weeks, five sessions will be given per week, and three sessions per week in the remaining 6 weeks. There are 28 sessions for each patient in total.

#### Control group

The acupuncture points aresham Tianshu (ST25), sham Fujie (SP14), sham Shangjuxu (ST37) (Additional file 2: Picture 2).

Sham location points are: about 2cm away from ST25, in the middle of the spleen and stomach channel; about 3 cm from SP14, in the middle of the spleen and stomach channel; one point beside ST37, in the middle of the stomach and gallbladder channel (Additional file 2: Picture 2).

The needle is inserted after sterilizing the skin by 1 to 1.5 cm, until the needle can be vertically fixed on the skin. No twirling lifting and thrusting manipulation is used. The sham electric stimulator (sham SDZ-V EA apparatus; Huatuo) is applied to the bilateral sham ST25 and sham SP14 with a dilatational wave of 10/50 Hz and electric current of 0.5 mA. The metal wire has been cut off inside to give the appearance of the real electric stimulator, with no current output. Length of treatment and the treatment sessions are the same as the treatment group.

#### Rescue medication

During the 2 weeks of screening and 12 weeks of follow-up, patients are asked to avoid any other treatments after 8 weeks of acupuncture treatment. An emergency treatment is permitted if participants do not have a bowel movement for three or more consecutive days during the trial -they are allowed to take 110 ml Glycerol Enema (Glycerol Enema, H11022362110, Beijing Maidihai Medical Co. Ltd; 110 ml/bottle) as rescue medication, which is provided by the investigators. Every use of Glycerol Enema should be recorded in the diary. And any other concomitant medications should also be recorded in the diary.

### Outcome measurement

The main endpoint is the weekly frequency of CSBMs, measured every week from weeks 1 to 20.

#### Primary outcome measures

The primary outcome of the study is the changed number of weekly average CSBMs during 8 weeks treatment compared with baseline.

#### Secondary outcome measures

The secondary outcome measures include the following eight items: 1) the proportion of participants having three or more CSBMs on average per week during the 20 weeks measured at the end of weeks 8 and 20 after randomization; 2) the changed number of weekly average spontaneous bowel movements (SBMs) during the 8 weeks of treatment measured at the end week 8; 3) stool consistency measured every week during the 8 weeks of treatment; 4) degree of difficulty in defecation measured every week during the 8 weeks of treatment; 5) patient assessment ofconstipationquality of life questionnaire (PAC-QOL) measured at the end of week 8 after randomization; 6) incidence of adverse events during the 8 weeks of treatmentmeasured at the end of week 8; 7) incidence of adverse events; 8) usage of medicine for constipation - any medicine for constipation used during the trial will be recorded, as well as the dosage and amount of every drug.

The participantis asked to keep a stool diary every day for 22 weeks, from 2 weeks before randomization to the end of week 20 after randomization. The content includes the SBM, CSBM, the stool consistency, degree of difficulty in defecation, the usage of drugs, diet, *etcetera*. The diaries will be collected once a week during the first 10 weeks, and every 4 weeks during the last 12 weeks.

All adverse events happening during the trial will be recorded, such as local hematoma, breaking of needle, retained needle after treatment, fainting, unbearable prickling, and continuous severe pain more than 1hour after acupuncture, local infection and abscess, *etcetera*. Serious adverse events should be reported to the principal investigator immediately.

### Quality control

To guarantee the quality of the study, the trial protocol is reviewed and revised by experts of acupuncture, gastroenterology, statistics, and methodology several times. A central randomization system is adopted to avoid selection bias. Strict eligible criteria are pre-set to restrict the research population. Blinded effect assessment and blinded statistics are designed to guarantee the objectivity of the data. All research staff, especially the acupuncture manipulators,are required to attend a series of training. The training courses include how to use the central randomization system, how to fill the case report form, how to manipulate interventions correctly, how to teach the patients to fulfil the diary, how to assess the effect, *etcetera*. A inspection plan is designed for quality inspecting. Monitors of 3 levels will check the 15 sub-centers regularly and finish paper which will be reported to the principal investigator. The first level is sub-center monitor, who appointed by the head of the sub-center; the second level is investigative hospital monitor, who appointed by Guang An Men Hosptila; the third level is the Quality Control Group monitor, who appointed by Evaluation Center of China Academy of Chinese Medical Scinece. Methods for improving compliance are also considered, such as traffic compensation, heath education, *etcetera*. A Data and Safety Monitoring Board (DSMB) was set up on 22 June 2012 to monitor the performance and safety of the trial, which is composed mainly of experts from different fields on the Chinese mainland.

### Sample size calculation and statistical analysis

According to our previous study, the weekly CSBM increase was conservatively estimatedat 1.49 times, with a standard deviation of 1.77, after 8 weeks EA treatment; the weekly CSBMs increased by 1.01, with a standard deviation of 1.61, after 8 weeks sham EA treatment. We used SAS 9.3 to define the 95% power, using a one-way analysis of variance fixed effects omnibus test, assuming a 20% loss to follow-up. To ensure the stratified randomization, the sample size calculation result is 1,034. As the main objective is to compare the changed number of weekly average CSBMs throughout the 8 weeks of treatment with the average weekly CSBMs during the 2 weeks baseline period, the primary analysis of the patient-level data is the number of CSBMs per week for 8 weeks. It means the total amount of CSBMs during 8 weeks will be divided by eight to achieve the result. (Each patient therefore has one number of weekly CSBMs throughout the 8 weeks of treatment). A simple way is to calculate the average number of CSBMs per week over 8 weeks for each patient and use non-parametric Wilcoxon statistics to test the null hypothesis that this number of CSBMs is the same between the EA and sham EA groups, with the alternative hypothesis that the change from baseline in CSBMs at 8weeks for the EA group does not equal the sham EA group. All patients at randomization are included in the analysis set regardless of whether they receive any treatment. According to the intent-to-treat principle, all analysis is based on the randomization analysis set. To account for correlation due to repeated measurements and to assess the dependence of the treatment effect on time, we will also use a random-effect Poisson regression model to compare the effect of EA versus sham EA on our primary outcome. If the distribution of the change from baseline in CSBMs at 8 weeks is a normal distribution, analysis of covariance (ANCOVA) will be used, and if the distribution is not a normal distribution, non-parametric analysis methods will be used. Study site and baseline will be included in the ANCOVA model as a covariant.

The secondary outcome measures include the following eight items: 1) the proportion of participants having three or more CSBMs on average per week; 2) the changed number of weekly average CSBMs during weeks 9 to 20; 3) the changed number of weekly average SBMs during the 8 weeks of treatment; 4) stool consistency; 5) degree of difficulty in defecation; 6) PAC-QOL; 7) incidence of adverse events; 8) usage of medicine for constipation. The stool consistency is classified into seven categories according to the Bristol Stool Scale [8]. A random effect ordinal regression model will be used to compare the difference in the stool consistency between the EA and sham EA groups. A random effect generalized linear model will be used to compare the difference in PAC-QOL between the EA and sham EA groups. All adverse events and serious adverse events will be listed. Random effect Poisson models will be used to compare the incidence of adverse events between the EA and sham EA groups. In addition, the usage of medicine for constipation will be compared between the two groups.

The subjects who donot receive any treatment after randomization or receive treatment but not any efficacy assessment data are classed as missing data, and an inverse probability weighting method will be used for multiple imputation to deal with the missing data.

Subgroup analysis will be conducted according to the subject’s age (<65 years and ≥65 years). In order to assess the central effect, the interaction effect of the study site and group will be tested.

A Fisher Exact test will be used to describe the case distribution in the EA group and the sham EA group and to calculate the total drop-out rate and the drop-out rate caused by adverse events between the two groups. Analysis of baseline characteristics will analyze the demographic data and other baseline measures. Mean and standard deviation will be used in the continuous variables an percentages in the categorical variables. For comparisons the two independent sample, *t*-test will be used for continuous variables and Chi-squared test for categorical variables. Non-parametric tests may also be used. A Chi-square test is used to analyze the difference in compliance between the two groups,to evaluate if the participants in the EA group and the sham EA group receive the specified treatment according to the protocol.

All statistical analyses will be two-sided tests. For all statistical analyses, SAS 9.1.3/SPSS Ver.13.0 software will be used. A *P* value of less than 0.05 (α-value of 0.05) is considered to indicate statistical significance. Mean and standard deviation will be used in the continuous variables and percentages in the categorical variables. For comparisons, the two independent sample *t*-test will be used for continuous variables and the Chi-squared test for categorical variables. Non-parametric tests will be used for variables which do not follow a normal distribution.

## Discussion

Functional constipation is one of the 43 common diseases for which acupuncture treatment is recommended by the World Health Organization [9]. Our earlier study showed that EA might be effective for functional constipation. Severe chronic functional constipation reduces the patients’ quality of life and causes some complications [10]. Medicine could increase the frequency of CSMBs and improve the quality of life, but it shows a short-term effect, which will mostly disappear after discontinuing medication and may give rise to side effects [11]. Our previous research indicated that EA is effective for functional constipation, especially having a post-treatment effect, but the result is insufficient to explain whether there was placebo effect. As the sample size was small, the superiority of EA for constipation could not be demonstrated clearly. This trial is designed to explore a standardized effective acupuncture method for severe chronic functional constipation and assess its efficacy and safety.

This study is supported by the 12th National Key Technology Support Program (No. 2012BAI24B01) which gives further financial support for functional constipation following the 11th National Key Technology Support Program. This trial is a multicenter, centrally randomized, controlled study which is large in scale (n=1034). Thus, quality control is considered as very important,as we described in the protocol.

To ensure the efficacy, we choose sham EA as a placebo. Some papers have reported that acupuncture has no significant differencescompared to sham acupuncture [12-14]. To confirm the efficacy of EA for severe chronic functional constipation, we chose a commonly used sham method as described in western journals as the control group - superficial insertion at non-acupuncture points [15] with no current output using an EA simulator. We used an EA apparatus which has the metal wire removed, giving an identical visual appearance to the real one. The electric stimulator looks normal but has no current output. This method helps with patient blinding, which should eliminate the placebo effect of EA as far as possible. Therefore, the results of this study could provide evidence to confirm the efficacy of EA for severe chronic functional constipation.

We must emphasize the choice of the main endpoint. Weekly frequency of CSBMs is the primary endpoint, which is considered to be clinically meaningful since it combines a subjective measure of the completeness of evacuation with an objective measure of the number of bowel movements and reflects the relief of chronic constipation [3].

This trial is also designed to observe the post-treatment effect of acupuncture during the follow-up period in weeks 4, 8 and 12 after treatment. The time this effect lasts for could indicate the effective superiority of EA. The secondary endpoint is the changed number of weekly average CSBMs during the follow-up stage, which could complement the comprehensive effects of acupuncture.

To ensure the quality and safety of our trial, a DSMB was set up which is composed mainly of experts from different fields in China. A DSMB secretariat was set up to service the board. All the DSMB experts have signed the ‘DSMB Consultant Agreement’ and participate in the regular meetings according to the DSMB Charter. Up to December 2012, two DSMB regular meetings have been held.

## Conclusion

This trial is expected to confirm that EA is effective and safe for severe functional constipation.

## Trial status

The first participants were included on 8 October 2012 and this article was submitted on 24 January 2013. To date, 285 participants have been recruited.

## Abbreviations

ANCOVA: Analysis of covariance; CSBM: Complete spontaneous bowel movement; DSMB: Data and Safety Monitoring Board; EA: Electro-acupuncture; PAC-QOL: Patient assessment of constipation quality of life questionnaire; SBM: Spontaneous bowel movement; TCM: Traditional Chinese Medicine.

## Competing interests

The authors declare that they have no competing interests.

## Authors’ contributions

ZL, JL, YZ, YC, LH, and BL participated in the conception and design of the trial, and drafting the manuscript. HX participated in the trial communication, monitoring. XZ and SY participated in the design of statistical analysis. LL is appointed as the DSMB chair for trial quality control. All the authors discussed, read, revised the manuscript, and all approved the publication of the protocol.

## Supplementary Material

Additional file 1 The acupuncture points of Acupuncture group. Click here for file

Additional file 2 The acupuncture points of Control group. Click here for file
