# Supplementary figures and images for: The efficacy and safety study of electro-acupuncture for severe chronic functional constipation: study protocol for a multicenter, randomized, controlled trial
Source: Trials. 2013 Jun 15;14:176. doi: 10.1186/1745-6215-14-176 (PMC3706209; doi:10.1186/1745-6215-14-176)

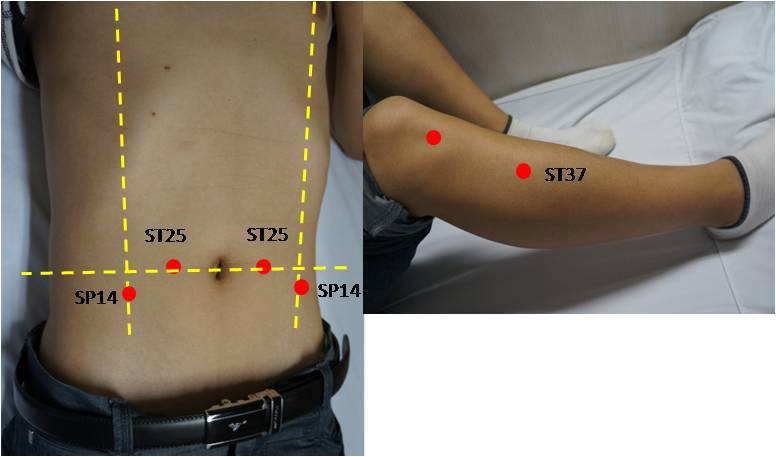

Supplement: Additional file 1 — The acupuncture points of Acupuncture group. [file 1745-6215-14-176-S1.jpeg]

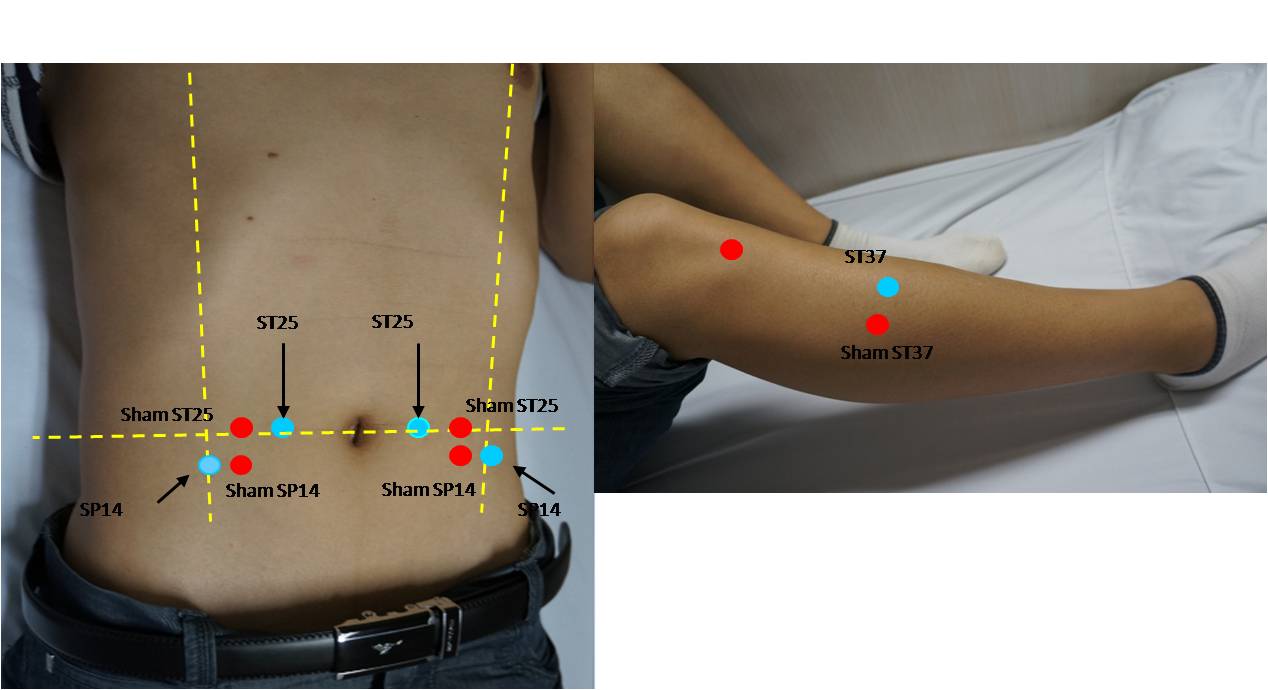

Supplement: Additional file 2 — The acupuncture points of Control group. [file 1745-6215-14-176-S2.jpeg]
